# Molecular Epidemiology of *Campylobacter* Isolates from Poultry Production Units in Southern Ireland

**DOI:** 10.1371/journal.pone.0028490

**Published:** 2011-12-06

**Authors:** Emer O'Mahony, James F. Buckley, Declan Bolton, Paul Whyte, Séamus Fanning

**Affiliations:** 1 UCD Centre for Food Safety, School of Public Health, Physiotherapy & Population Science, UCD Veterinary Sciences Centre, University College Dublin, Belfield, Dublin, Ireland; 2 Veterinary Food Safety Laboratory, Cork County Council, Inniscarra, County Cork, Ireland; 3 Food Safety Department, Teagasc Food Research Centre, Ashtown, Dublin, Ireland; Wageningen University and Research Centre, The Netherlands

## Abstract

This study aimed to identify the sources and routes of transmission of *Campylobacter* in intensively reared poultry farms in the Republic of Ireland. Breeder flocks and their corresponding broilers housed in three growing facilities were screened for the presence of *Campylobacter* species from November 2006 through September 2007. All breeder flocks tested positive for *Campylobacter* species (with *C. jejuni* and *C. coli* being identified). Similarly, all broiler flocks also tested positive for *Campylobacter* by the end of the rearing period. Faecal and environmental samples were analyzed at regular intervals throughout the rearing period of each broiler flock. *Campylobacter* was not detected in the disinfected house, or in one-day old broiler chicks. *Campylobacter jejuni* was isolated from environmental samples including air, water puddles, adjacent broiler flocks and soil. A representative subset of isolates from each farm was selected for further characterization using *flaA*-SVR sub-typing and multi-locus sequence typing (MLST) to determine if same-species isolates from different sources were indistinguishable or not. Results obtained suggest that no evidence of vertical transmission existed and that adequate cleaning/disinfection of broiler houses contributed to the prevention of carryover and cross-contamination. Nonetheless, the environment appears to be a potential source of *Campylobacter*. The population structure of *Campylobacter* isolates from broiler farms in Southern Ireland was diverse and weakly clonal.

## Introduction

*Campylobacter* continues to be the most commonly reported cause of bacterial gastroenteritis in the European Union (EU). In total there were 198,252 confirmed cases of campylobacteriosis in 2009, giving an overall crude incidence rate (CIR) of 45.6 per 100,000 population [1].

While a range of risk factors for infection with *Campylobacter* have been identified, the most common is the handling and/or consumption of undercooked poultry, in particular chicken. According to a recent opinion of the European Food Safety Authority's (EFSA) Biological Hazards (BIOHAZ) Panel, 50 to 80% of human cases of campylobacteriosis may be attributed to the chicken reservoir [2]. This report also outlined the considerable underreporting of cases of campylobacteriosis and suggested that no less than 2 million and up to 20 million cases of clinical campylobacteriosis occur per year in the EU.

In 2008, a baseline survey on *Campylobacter* in broiler batches and carcasses in the EU was undertaken [3]. The prevalence at community level of *Campylobacter*-colonized broiler batches was reported to be 71.2% and the prevalence of *Campylobacter*-contaminated broiler carcasses was found to be 75.8%. The colonization of broiler flocks with *Campylobacter* is therefore a significant food safety issue and a reduction in the number of contaminated poultry products entering the food chain would reduce the negative impact on public health.

It is thought that the reduction of *Campylobacter*-contaminated poultry meat can be achieved most effectively by implementing on-farm control measures [4].

Extensive research into the most important source of *Campylobacter* in poultry production units has been carried out. Vertical transmission has previously been implicated [5], [6], [7], [8]. Carryover to subsequent flocks as a result of inadequate disinfection of broiler houses has also been identified as a risk factor [9]. Horizontal transmission from the surrounding environment to the broiler house either *via* the farm workers or other vectors such as wild birds, vermin and house flies, is considered to be the most likely source of contamination [10], [11]. Risk factors for infection may vary from country to country because of differing farming practices and associated climatic conditions. Therefore, efforts to understand the relative importance of each potential source and transmission route of *Campylobacter* infection on-farm, continues to have an important role in extending our understanding of the epidemiology of this important pathogen.

In 2009, there were 1,808 confirmed cases of campylobacteriosis in Ireland [12]. The prevalence of *Campylobacter*-colonized broiler batches in Ireland in 2008 was 83.1% and of that *Campylobacter*-contaminated broiler carcasses accounted for 98.3% [3]. In order to implement effective control measures and reduce the prevalence of *Campylobacter* in Irish poultry products, the most significant transmission routes must be identified. The aim of this study was to investigate the occurrence of *Campylobacter* in a subset of intensively reared Irish poultry flocks and to identify sources of *Campylobacter* in each farm environment. Molecular sub-typing methods were used to identify the genotypes present in Irish broiler farms and to shed further light on possible transmission routes of *Campylobacter* in poultry farms.

## Materials and Methods

### Description of Farms in this Study

Three housed broiler flocks (denoted as broiler flocks 1, 2 and 3), located on three different farms (denoted as broiler farms 1, 2 and 3) in different geographical locations of Ireland, were studied throughout their 6–7 week life span. The flocks were screened between November 2006 and September 2007. Farm 1 consisted of three poultry house units sampled from November 2006 to January 2007. Farm 2 consisted of two poultry house units that were sampled between April 2007 and May 2007. Farm 3 consisted of one poultry house unit and was sampled between July 2007 and September 2007. Broiler flock sizes ranged from approximately 18,000 to 34,000 birds per house. Three breeder flocks, located on breeder farm 1 (denoted as breeder farm 1, flocks 1–3), supplied chicks for broiler farm 1. Broiler farm 2 was supplied by breeder farms 2 and 3. Broiler farm 3 was supplied by two flocks from breeder farm 1 (denoted as breeder farm 1, flocks 4 and 5), and one flock from both breeder farms 4 and 5. Each breeder flock comprised approximately 5,000 birds per house.

### Sample Collection

Samples were collected approximately every 14 days from (i) the cleaned and disinfected broiler houses prior to chick placement, (ii) the chickens and (iii) the environments inside and outside the broiler houses. The breeder flocks supplying each broiler house were also tested. Samples were transported to the laboratory at 4°C in a cool box and processed on the same day.

#### Breeder flocks

Sixty fresh faecal samples (5 pooled faecal samples each containing 12 fresh faecal droppings) were collected from the floor of the house of the corresponding breeder farms supplying broiler hatching eggs for each flock in this study.

#### Broiler flocks

One-day old broiler chicks were tested by enriching the faeces-soiled paper that lined the crates used to transport the birds from the hatchery to the broiler house (100 birds per crate). Faecal samples were then taken at regular intervals throughout the rearing period of each flock (at days 14, 28 and 42 approximately). Sixty individual fresh faecal samples were collected from the broiler house floor and combined (as outlined previously).

#### Environmental samples

Samples from walls, floors, structural support columns, feed and water dispensers along with concrete aprons (the concreted area outside the front of the house) were taken using sterile swabs pre-moistened with 10 ml Maximum Recovery Diluent (MRD, Lab M Ltd., Bury, UK). An area of 0.1 m^2^ of the object's surface was chosen for sampling, and swabbing continued outside this area until either the entire surface was sampled or the swab was dry. Two litres of water supplying the broiler house drinkers were also taken. Aspiration of air samples onto one *Campylobacter* blood-free selective agar base (CCDA, CM0739, Oxoid, Cambridge, UK), supplemented with cefoperazone and amphotericin B (CCDA selective supplement) (Oxoid), and one Tryptone Soya Agar (TSA, Oxoid Ltd., Cambridge, UK) plate was performed using a Sampl'air MK2 double agar plate sampler (AES Laboratoire Groupe, Combourg, France). Two air samples (500 l×2) were taken from inside and one directly outside each broiler house on each sampling occasion (at days -1, 14, 28 and 42). Occasionally, samples were taken opportunistically inside and outside the broiler house environment (Table 1).

**Table 1:** *Campylobacter* sources during the sampling period across farms.

Flock	Sample	Number of positive samples/number of samples tested				
		Day -1^*^	Day 1	Day 14^*^	Day 28^*^	Day 42^*^
**1**	Broilers	-	0/48^c^	**3/5** ^c^	**5/5** ^c^	**5/5** ^c^
	Adjacent Broiler 1	-	-	0/2^c^	**2/2** ^c^	**2/2** ^c^
	Adjacent Broiler 2	-	-	**2/2** ^c^	**2/2** ^c^	-
	Air	0/6	-	**1/6**	0/6	0/6
	Puddle	0/1	-	**1/2**	0/1	0/2
	Soil	0/2	-	0/2	0/2	**1/2**
	Other	0/20^ap, cl co, d, f, fe, fl, h, w, wa^	-	0/7^ap, h, i, wa^	0/9^ap, h, i, wa^	0/11^ap, h, i, wa^
**2**	Broilers	-	0/48^c^	0/5^c^	0/5^c^	**5/5** ^c^
	Adjacent Broiler	-	-	0/2^c^	0/2^c^	-
	Soil	0/2	-	0/2	**1/2**	0/2
	Other	0/22^a, ap, co, d, fe, fl, p, w, wa^	-	0/10^a, ap, wa^	0/13^a, ap, i, wa^	0/12^a, ap, p, r, wa^
**3**	Broilers	-	0/48^c^	0/5^c^	**5/5** ^c^	**5/5** ^c^
	Soil	0/2	-	0/2	0/2	**1/2**
	Other	0/24^a, ap, cl co, d, fe, fl, p, w, wa^	-	0/12^a, ap, p, wa^	0/12^a, ap, p, wa^	0/10^a, ap, wa^

Bold type indicates positive result; -, not tested; *, approximate day of sampling; ^a^, air; ^ap^, apron; ^cl^, clothing; ^co^, support columns; ^d^, drinkers; ^f^; fan, ^fe^; feeder, ^fl^; floor, ^h^; horse faeces; ^i^, insects; ^p^, puddles; ^r^, rodent faeces; ^w^, walls; ^wa^, water; ^c^, composite samples.

### Isolation of *Campylobacter*

To determine the presence/absence of *Campylobacter*, samples were examined by direct plating and/or enrichment culture methodologies based on the Horizontal Method for Detection and Enumeration of *Campylobacter* spp. (ISO 10272-1:2006) and the Detection and Semi-quantitative Enumeration of Thermotolerant *Campylobacter* spp. (ISO 17995:2005). Isolation of emerging *Campylobacter* species, based on the method previously described [13], was also performed for faecal samples. Following incubation, five suspect colonies were randomly selected from plates and subcultured to obtain pure colonies.

For the lined transport crates, 60 papers from each flock were collected at random and divided into six piles consisting of ten papers on top of one another. Each pile of ten papers was then aseptically cut into 8 strips. Each strip was enriched in 200 ml *Campylobacter* Enrichment Broth (CEB, Lab M Ltd., Bury, UK) supplemented with 5% (v/v) lysed horse blood (TCS Biosciences, Buckingham, UK) and cefoperazone, vancomycin, trimethoprim and cyclohexamide (CVTC supplement, Lab M Ltd., Bury, UK). Forty-eight composite samples, each consisting of ten strips, from each flock were tested in this way. Swabs were enriched in 100 ml of CEB supplemented with 5% (v/v) lysed horse blood and CVTC. Water samples were filtered using 0.45 µm filters (Millipore, Billerica, MA., USA), which were then enriched in sterile 30 ml containers containing 20 ml of CEB supplemented with 5% (v/v) lysed horse blood and CVTC. For the air samples, the agar from each TSA plate was aseptically removed and enriched in 100 ml CEB supplemented with 5% (v/v) lysed horse blood and CVTC, while each CCDA plate was incubated directly. Faecal samples from horses, crushed flies and beetles were enriched with CEB supplemented with 5% (v/v) lysed horse blood and CVTC using a 1∶10 ratio of sample to broth. Water from puddles was enriched with an equivalent volume of double-strength CEB supplemented with 5% (v/v) lysed horse blood and CVTC.

### Biochemical confirmation

Presumptive *Campylobacter* isolates were confirmed using standard biochemical procedures including a Gram lysis test (3% [w/v] KOH, Sparks Lab Supplies, Dublin, Ireland), oxidase test (Oxoid, Cambridge, UK), catalase test (3% H_2_O_2_, Sigma Aldrich, St. Louis, MO, USA) and l-alanine aminopeptidase test (Aminopeptidase Test, Fluka Biochemika, Buchs, Switzerland). A latex agglutination test was also used (DrySpot Campylobacter Test Kit, Oxoid, Cambridge, UK). Following biochemical confirmation, isolates were stored at −20 and −80°C on Protect cryobeads (Technical Service Consultants Ltd., Heywood, Lancashire, UK) containing 80% [v/v] glycerol, for further analysis.

### Genotyping

#### Multiplex Polymerase Chain Reaction (mPCR)

DNA purification was carried out using a commercial kit (Wizard Genomic DNA Purification Kit, Promega, Madison, WI, USA). Isolates were identified to species level as described previously [14]. Amplicon sizes were determined by comparison with a molecular weight marker (50 bp ladder, Promega) following migration on a 1.5% [w/v] agarose gel.

#### flaA-SVR sequencing

Sequencing of an internal 321-bp fragment of the flagellin A short variable region (SVR), was performed using the primer pair Fla242FU [5′-CTA TGG ATG AGC AAT T (AT) A AAAT-3′] and Fla625RU [5′-CAA G (AT) C CTG TTC C (AT)A CTG AAG-3′], as previously described by Meinersmann *et al.* [15]. All *flaA*-derived amplicons were purified using a QIAquick PCR Purification kit (Qiagen GmbH, Hilden, Germany) and sequenced commercially by MWG Biotech (Ebersberg, Germany). The nucleotide sequences were deposited in the internet accessible *flaA*-SVR sequence database (http://hercules.medawar.ox.ac.uk/flaA/) and SVR allele numbers were assigned by sequence comparisons against the existing *flaA*-SVR sequences. The DNA trace files were submitted for confirmation of novel *flaA*-SVR alleles.

#### Multi Locus Sequence Typing (MLST)

Internal fragments of seven gene targets were amplified by PCR and their nucleotide sequences determined with primers and reaction conditions in accordance with the published MLST scheme for *C. jejuni* and *C. coli*. Allele identification followed by sequence type (ST) and clonal complex (CC) assignments were done by interrogation of the *Campylobacter* MLST database for each isolate (http://pubmlst.org/campylobacter).

### Data Analysis

Trimmed *flaA*-SVR sequences (including STs) were imported into BioNumerics, Version 6.1 (Applied Maths, Sint-Martens-Latem, Belgium). A dendrogram was created using the unweighted-pair group method with arithmetic mean (UPGMA).

## Results

### Presence of *Campylobacter* on poultry farms

#### Broiler breeders

All broiler breeder flocks supplying each rearing farm were found to be colonized with both *C. jejuni* and *C. coli* (Table 2). A total of 27 *C. jejuni* and 19 *C. coli* were isolated from breeder farm 1 (containing flocks 1, 2 and 3). Two *C. jejuni* and 42 *C. coli* were isolated from breeder farm 2. One *C. coli* isolate and 22 *C. jejuni* isolates were recovered from breeder farm 3. There were 6 *C. jejuni* and 14 *C. coli* isolates recovered from breeder farm 1, flock 4, along with 7 *C. jejuni* and 12 *C. coli* from breeder farm 1, flock 5. A total of 13 *C. jejuni* and 7 *C. coli* were isolated from breeder farm 4, while 26 *C. jejuni* and 25 *C. coli* were recovered from breeder farm 5.

**Table 2:** *Campylobacter* species recovered from all farms tested.

Farm	Day	Source	Sample	Species (no.)
1	-	Breeder 1 Flock 1	Faeces	*C. jejuni* (6), *C. coli* (2)
	-	Breeder 1 Flock 2	Faeces	*C. jejuni* (6), *C. coli* (9)
	-	Breeder 1 Flock 3	Faeces	*C. jejuni* (15), *C. coli* (8)
	13	Broiler	Faeces	*C. jejuni* (10)
	13	Adjacent Broiler 2	Faeces	*C. jejuni* (3)
	13	Environment Inside	Air	*C. jejuni* (2)
	13	Environment Outside	Puddle	*C. jejuni* (1)
	32	Broiler	Faeces	*C. jejuni* (9)
	32	Adjacent Broiler 1	Faeces	*C. jejuni* (1)
	32	Adjacent Broiler 2	Faeces	*C. jejuni* (8)
	42	Broiler	Faeces	*C. jejuni* (26)
	42	Adjacent Broiler 1	Faeces	*C. jejuni* (5)
	42	Environment Outside	Soil	*C. jejuni* (1)
**Total**				*C. jejuni* (93) *C. coli* (19)
2	-	Breeder 2	Faeces	*C. jejuni* (2), *C. coli* (42)
	-	Breeder 3	Faeces	*C. jejuni* (22), *C. coli* (1)
	27	Environment Outside	Soil	*C. jejuni* (5)
	41	Broiler	Faeces	*C. jejuni* (24)
**Total**				*C. jejuni* (53) *C. coli* (43)
3	-	Breeder 4	Faeces	*C. jejuni* (13), *C. coli* (7)
	-	Breeder 5	Faeces	*C. jejuni* (26), *C. coli* (25)
	-	Breeder 1 Flock 4	Faeces	*C. jejuni* (6), *C. coli* (14)
	-	Breeder 1 Flock 5	Faeces	*C. jejuni* (7), *C. coli* (12)
	27	Broiler	Faeces	*C. jejuni* (23)
	41	Broiler	Faeces	*C. jejuni* (26)
	41	Environment Outside	Soil	*C. jejuni* (1)
**Total**				*C. jejuni* (102) *C. coli* (58)

-, not applicable.

#### Broiler chickens

*Campylobacter* could not be cultured from the transport crate paper liners on the day the chicks arrived at the rearing house. All three broiler flocks were found to be contaminated with *Campylobacter* by the end of the rearing period. *Campylobacter jejuni* was the only species isolated from all three poultry flocks under investigation (Table 2). Faecal samples from broiler flock 1 were contaminated with *Campylobacter* on days 13, 32 and 42. Faecal samples from broiler flock 2 were negative until the final sampling day (day 41). Broiler flock 3 was found to be contaminated with *Campylobacter* on days 27 and 41.

#### Presence of Campylobacter in the poultry farm environment

On all three farms, *Campylobacter* could not be detected in the empty poultry house structure/environment after the cleaning and disinfection procedure had been carried out. All samples taken from the external environment of the cleaned house, including soil, air, the concrete apron and horse faeces were negative for *Campylobacter* (Table 1).

During the rearing period of each flock, the environment was found to be contaminated with *Campylobacter* (Table 1). In the case of broiler farm 1 (day 13), an air sample taken inside the rearing house, an adjacent broiler flock and a puddle outside the house were sampled and found to be contaminated with *Campylobacter*. On day 32, two adjacent broiler flocks tested positive for *C. jejuni*. On day 42, one adjacent broiler house had been depopulated. *Campylobacter jejuni* was isolated from the remaining adjacent flock and was also recovered from an environmental soil sample. In the case of broiler farm 2, *C. jejuni* was isolated from a soil sample taken on day 27. On broiler farm 3, *C. jejuni* was recovered from a soil sample taken on day 41.

### Molecular sub-typing and spatio-temporal tracking of *Campylobacter* isolates

**Figure 1:**
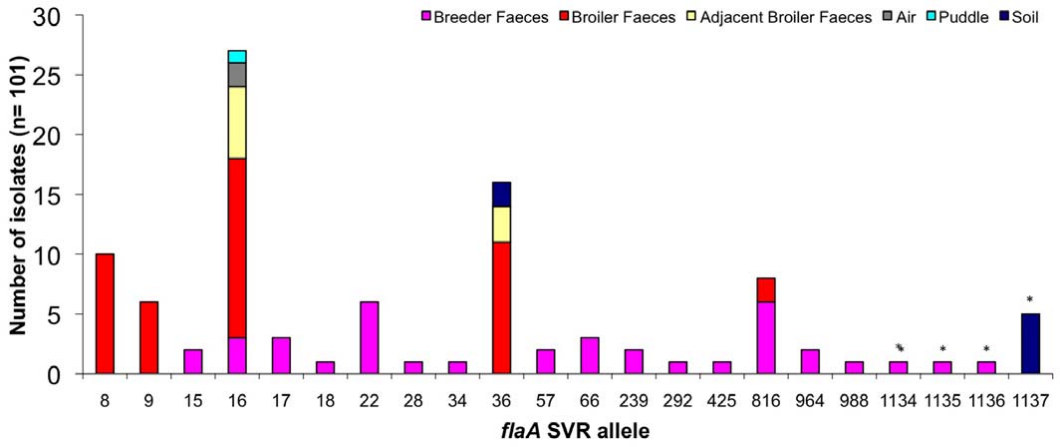
Distribution of *flaA*-SVR alleles detected according to source, (asterisks signify new *flaA*-SVR alleles).

A representative subset of *Campylobacter* isolates was chosen from each farm and characterized by DNA sequence analysis of the SVR containing region of the *flaA* gene (*flaA*-SVR), to determine if same-species isolates from different sources could be distinguished. Figure 1 shows the distribution of allele types discovered in relation to the source of each isolate. A total of 22 *flaA*-SVR alleles were identified in the 101 isolates investigated.

**Figure 2:**
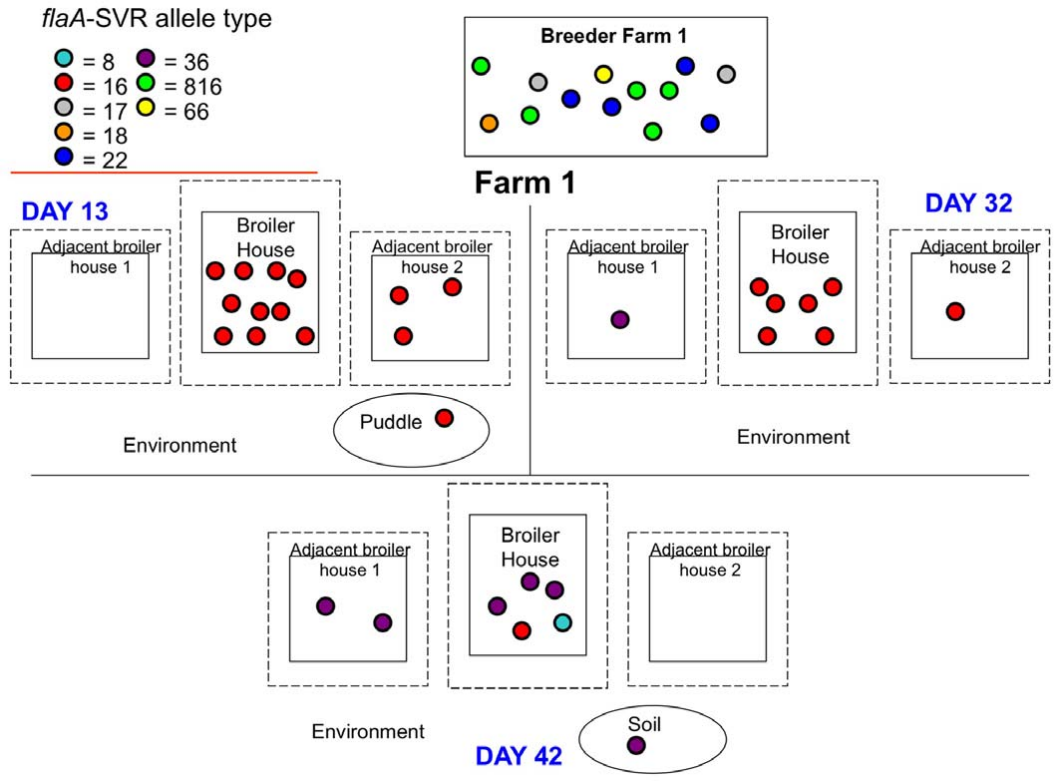
Schematic diagram of the *flaA*-SVR alleles detected on farm 1.

**Figure 3:**
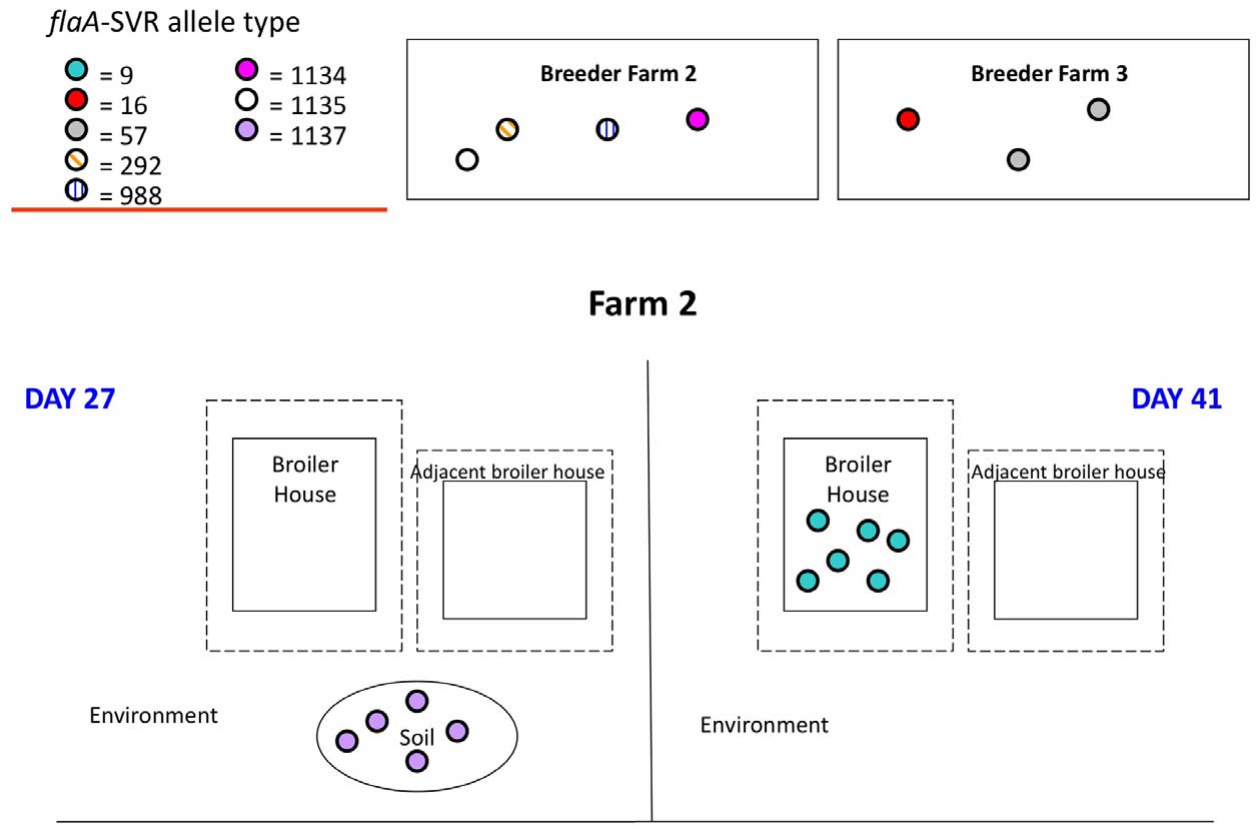
Schematic diagram of the *flaA*-SVR alleles detected on farm 2.

**Figure 4:**
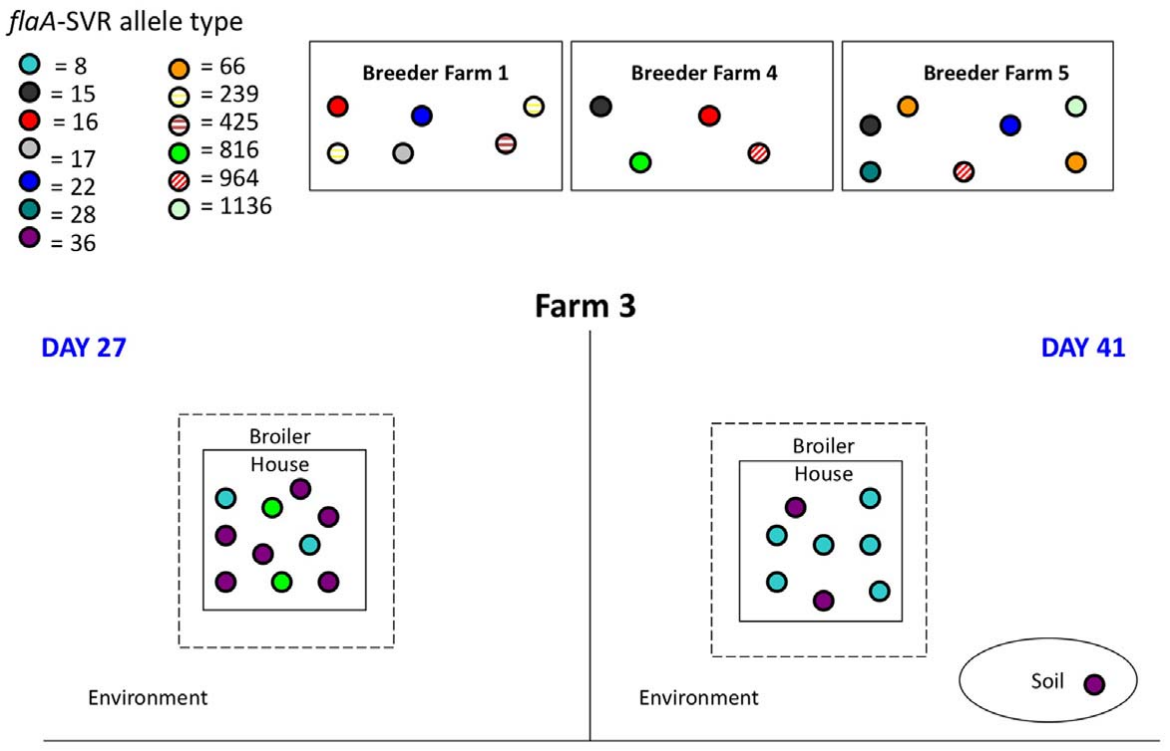
Schematic diagram of the *flaA*-SVR alleles detected on farm 3.

Figures 2, 3 and 4 show the spatio-temporal distribution of *flaA*-SVR alleles identified on each sampling day across each of the three broiler farms. This sub-typing analysis revealed a diverse and weakly clonal population structure of *C. jejuni*, with multiple subtypes present throughout the lifecycle of each flock. *Campylobacter* isolates originating from each set of breeders and the faeces from their respective progeny presented with non-identical *flaA*-SVR DNA sequences.

In the case of farm 1, *flaA*-SVR allele number 16 was detected on day 13 in faeces from the broiler flock, faeces from adjacent broiler flock 2, and in a puddle located outside the house (Figure 2). On day 32, allele number 16 continued to be identified in the broiler and adjacent broiler flock 2 faeces. On the same day, allele number 36 was detected in faeces from adjacent broiler flock 1. On day 42, allele number 36 could be detected in faeces from adjacent broiler flock 1, and was also now identified in broiler flock 1 and in an environmental soil sample. Allele numbers 8 and 16 were also detected in the broiler faeces.

In the case of farm 2, allele number 1137 was identified in an environmental soil sample taken on day 27 (Figure 3). *Campylobacter* was not isolated from broiler flock 2 faeces until the final sampling day, when allele number 9 was detected.

In the case of farm 3, allele numbers 8, 36 and 816 were detected in the broiler faeces on day 27 (Figure 4). On day 41, alleles 8 and 36 were again identified. Allele 26 was also found to be present in a soil sample outside the house on this day.

### Diversity of MLST Sequence Types (STs) and Clonal Complexes (CCs)

**Figure 5:**
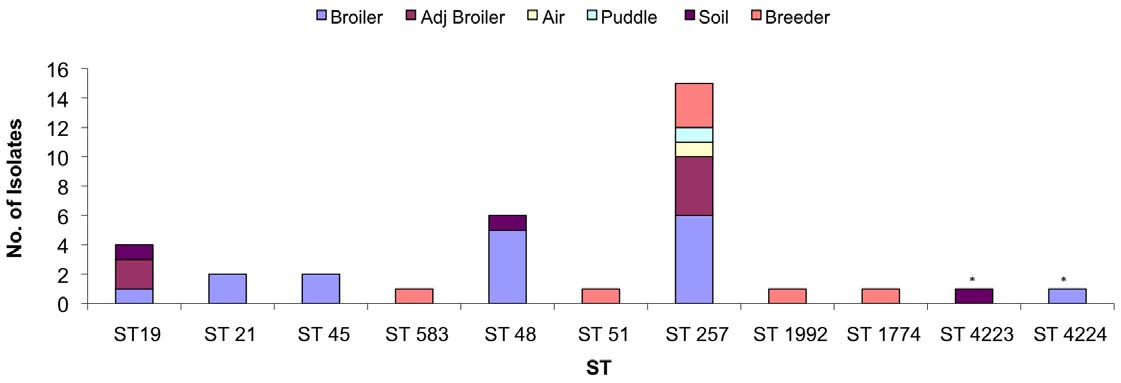
Distribution of STs detected according to source (asterisks signify new STs).

In order to substantiate *flaA*-SVR findings, a subset of isolates from each farm was chosen for MLST analysis.Eleven STs were identified among the 35 isolates (34 *C. jejuni*, 1 *C. coli*) chosen. Five STs were assigned to more than one isolate while six STs were assigned to single isolates. Figure 5 shows that ST 257 was the most frequently detected sub-type (in 15/35 isolates), followed by ST 48 (in 6/35 isolates) and ST19 (in 4/35 isolates). ST 21 and ST 45 were assigned to two isolates each. ST 583, ST 51, ST 1922, ST 1744, ST 4223 and ST 4224 were assigned to single isolates. Two novel sequence types (ST 4223 and ST 4224) were identified and submitted to the *C. jejuni* MLST database (http://pubmlst.org/campylobacter/).

**Figure 6:**
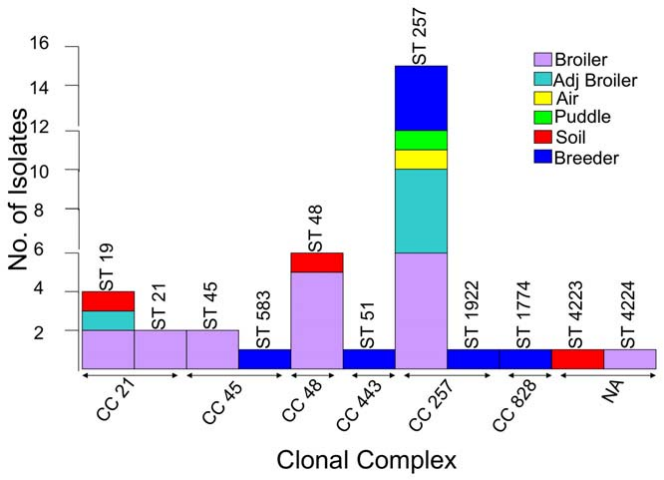
Distribution of CCs and STs according to source.

All 11 STs were grouped into 6 CCs. The two novel sequences could not be grouped into a defined CC. The largest CC was found to be CC 257 (consisting of 16 isolates), followed by CC 21 and CC 48 (6 isolates each). In total, 80% (28/35) of isolates analyzed by MLST were grouped into one of these three CCs. Isolates grouped in CC 257 were found to have originated from a variety of sources (Figure 6).

**Figure 7:**
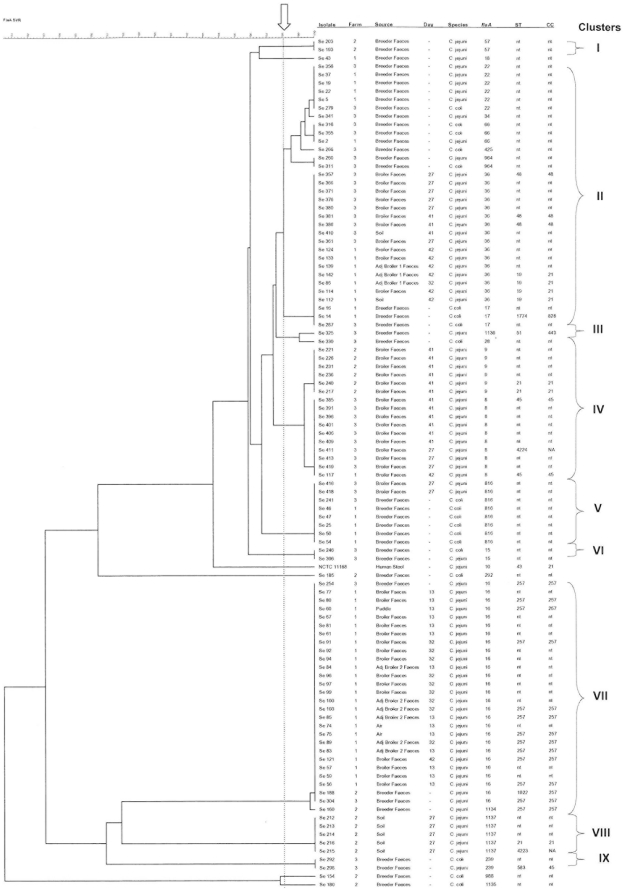
Comparison of *flaA*-SVR sequences of *Campylobacter* isolates from breeder and broiler farms and farm environments. A total of 101 isolates were included. Strain *Campylobacter jejuni* NCTC11168 was included as a control strain in the pairwise analysis. The arrow indicates the 98% similarity cut-off point. *flaA*, *flaA*-SVR allele number; ST, sequence type; CC, clonal complex; Nt, not tested; NA, not assigned; -, not applicable.

Figure 7 depicts the phylogenetic relationship between the 22 *flaA*-SVR types and the 11 STs identified during the study. Nine cluster genotypes were observed using a threshold genetic similarity of 98% as a cut-off coefficient value.

## Discussion

Despite extensive research, the definitive sources of infection and routes of transmission of *C. jejuni* in the poultry reservoir remain to be fully elucidated.

The purpose of this study was to identify sources of *Campylobacter* in intensively reared broiler flocks in Ireland.

Molecular sub-typing techniques have previously shown the population structure of *C. jejuni* to be highly diverse and weakly clonal [16], [17]. This study confirmed that genetic diversity also exists among Irish *C. jejuni* strains isolated from broiler flocks, adjacent flocks and the farm environment.

The occurrence of vertical transmission of *Campylobacter* in poultry has been a controversial issue. The isolation of *Campylobacter* species from the reproductive tract of broiler breeders has been reported [6], [7] and transmission from breeder hens to broiler chickens has been suggested [18]. Conflicting reports have also been published suggesting that vertical transmission is unlikely or of little importance [19], [20]. In this study, while breeder flocks were found to be colonized with *Campylobacter*, molecular characterization confirmed the isolates to be of different *flaA*-SVR and ST types, suggesting that vertical transmission was not involved in the colonization of these broiler flocks with *Campylobacter*. This work is in agreement with a related study conducted by Patriarchi *et al.*, where none of the genotypes identified in breeder flocks were subsequently identified on any of the broiler farms [21]. Colonization of chickens with *Campylobacter* usually occurs between 3 and 5 weeks of age, and once infected, prevalence in a flock can often be close to 100% [22]. Interestingly, *Campylobacter* was not detected in any of the three broiler flocks in this study before day 13 of the rearing cycle. A possible factor contributing to the delay in colonization of chickens with *Campylobacter* is the presence of protective maternal antibodies in young chicks [23], [24].

In this study, *Campylobacter* was not isolated from broiler flock 2 until after the process of partial depopulation or thinning had been carried out. The practice of thinning has previously been reported as an important risk factor for *Campylobacter* colonization of residual birds [25], [26], [27]. It has been shown *in vitro* that the presence of the neurotransmitter noradrenaline stimulates the growth and motility of *C. jejuni* [28]. As a result of triggering the release of noradrenaline, the stressful thinning process could be expected to contribute to rapid growth of the bacterium in the avian gastrointestinal tract leading to increased shedding of *Campylobacter* by birds, and the subsequent rapid spread of the bacteria.

Challenges in maintaining biosecurity during the thinning process can result in cross-contamination from environmental sources. Allen *et al.* reported the isolation of *Campylobacter* from transport vehicles, equipment, personnel and the farm driveways prior to the thinning process [25]. Using *flaA*-SVR and MLST subtyping methods, Patriarchi *et al.* also identified the practice of partial depopulation as a potential source and route of flock contamination on Irish broiler farms. Molecular evidence of the role of transport crates in introducing *Campylobacter* spp. into the broiler house was also reported [21]. In the case of farm 1 in this study, a *C. jejuni* isolate identified as *flaA*-SVR allele type 36 was isolated from an adjacent broiler flock prior to thinning. On the final sampling day, this *flaA*-SVR allele type was again isolated in the adjacent broiler flock, and was also identified in broiler flock 1 and in an environmental soil sample. Improved biosecurity measures in relation to the depopulation process may contribute to the prevention or delayed colonization of chickens with *Campylobacter*.

Previous studies have reported that poultry strains are frequently found to be genetically distinct from environmental isolates [29], [30]. In the case of broiler farm 2, *C. jejuni flaA*-SVR allele types isolated from broilers and environmental soil samples were confirmed as non-identical. However, identical *C. jejuni* strains were identified from air, soil, water puddles and chickens on broiler farms 1 and 3, suggesting that transfer of campylobacters between these environments may be occurring. However, it is not possible based on the epidemiological data presented here to establish the direction of a given exchange. Bi-directional movement of *Campylobacter* between sources cannot be ruled out and has been implicated previously [31]. In the case of two broiler flocks studied here, contamination of the farm environment was not detected until after the chickens had become infected, highlighting the broilers as a possible source of environmental contamination. MLST data from flock 1 further demonstrate that genetically identical strains can be isolated from broiler faeces and environmental samples. On day 13, ST 257 was found to be present in 2 broiler houses and in a water puddle outside the house. This ST was identified in the broiler flock on all sampling days. On day 42, ST 19 and ST 45 were also detected. These results reflect the findings of previous spatio-temporal studies on broiler farms, where different STs were identified in chicken faeces as the rearing period progressed [11].

During 2007, New Zealand experienced a 50% decline in the rate of campylobacteriosis notifications and hospitalisations [32]. This decline followed the introduction of voluntary and regulatory interventions to reduce contamination of poultry with *Campylobacter* species and was sustained in 2008 and 2009. A number of other countries have reported a reduced incidence of campylobacteriosis infections following the implementation of poultry-focussed control strategies [33], [34], [35], [36]. Various interventions were employed in each country however all strategies included strengthening on-farm biosecurity and monitoring the prevalence of *Campylobacter*-positive flocks. The implementation of similar measures in poultry farms in Ireland could contribute to a reduction in human campylobacteriosis infection rates and lead to improved public health protection.

The population structure of Campylobacter isolates from broiler farms in Southern Ireland was determined, (based on these data), to be weakly clonal. Such genetic diversity complicates the challenge of managing Campylobacter species population dynamics within the poultry farm environment. Nevertheless, our data point to the importance of applying more than one sub-typing method as part of our epidemiological studies to carefully describe this dynamic process.

It is reasonable to conclude that there are multiple sources from which *Campylobacter* can be transmitted on broiler farms. Following their introduction into broiler flocks, the spread of *Campylobacter* is influenced by various host and environmental factors, such as biosecurity measures in place, farming practices, the immune status of the chickens, and the presence of other animals on the farm. A combined protocol individually targeting each potential source and route of transmission is required as a logical approach to effectively reduce the colonization of broiler chickens with *Campylobacter* in a step-wise fashion.
